# Autoimmune hepatitis following long-term denosumab therapy in a patient with Hashimoto thyroiditis

**DOI:** 10.1210/jcemcr/luag251

**Published:** 2026-10-16

**Authors:** Jacob Maniattu, McAnto Antony, Alvin Maliakal

**Affiliations:** Department of Internal Medicine, Eastern Virginia Medical School at Old Dominion University, Norfolk, VA 23507, USA; Department of Internal Medicine, Medical University of South Carolina/AnMed Campus, Anderson, SC 29621, USA; Department of Internal Medicine, Eastern Virginia Medical School at Old Dominion University, Norfolk, VA 23507, USA

**Keywords:** denosumab, autoimmune hepatitis, RANK ligand inhibitor, drug-induced liver injury, osteoporosis, adverse drug reaction

## Abstract

Denosumab, a receptor activator of nuclear factor-κB ligand (RANKL) inhibitor, is widely used to treat osteoporosis. Its role as a trigger for autoimmune hepatitis (AIH) remains uncertain. We report a 68-year-old woman with osteoporosis and Hashimoto thyroiditis who developed jaundice and severe hepatocellular liver injury after 3 years of denosumab therapy. Alternative viral, obstructive, and metabolic causes were excluded. Liver biopsy demonstrated interface hepatitis with a lymphoplasmacytic portal infiltrate consistent with AIH, and autoimmune serologies were positive. Hemophagocytic lymphohistiocytosis was excluded, and an International AIH Score of 17 supported a diagnosis of definite AIH. Treatment with corticosteroid-based immunosuppression resulted in complete biochemical remission, and denosumab was discontinued with transition to zoledronic acid to prevent rebound bone loss. Whether this case represents drug-induced autoimmune-like hepatitis or de novo idiopathic AIH occurring in a susceptible individual cannot be determined definitively. Clinicians should maintain a low threshold for evaluating liver injury in patients receiving denosumab who develop jaundice or marked transaminase elevation.

## Introduction

Denosumab is a fully human monoclonal antibody targeting the receptor activator of nuclear factor-κB ligand (RANKL), blocking osteoclast activity and inhibiting bone resorption. It is widely used for osteoporosis and bone complications of malignancy. Hepatotoxicity is not listed in the United States Food and Drug Administration-approved labeling for any denosumab product, and routine liver monitoring is not recommended [1].

Autoimmune hepatitis (AIH) is a chronic, immune-mediated inflammatory liver condition presenting with elevated transaminases, hypergammaglobulinemia, and positive autoantibodies, including antinuclear antibodies, anti-smooth muscle antibodies, or liver-kidney microsomal antibodies. Histology demonstrates lymphoplasmacytic infiltrate with interface hepatitis [2]. Notably, Hashimoto thyroiditis is the most common concurrent autoimmune disease in type 1 AIH, present in 10% to 23% of cases [3].

Drug-induced autoimmune-like hepatitis (DI-ALH) shares features with idiopathic AIH but typically presents within 3 to 12 months of drug initiation. Denosumab has been implicated in only a small number of published hepatotoxicity cases [4, 5]. We present a case of severe AIH arising after 3 years of denosumab therapy and discuss the diagnostic challenge of distinguishing DI-ALH from de novo idiopathic AIH in a patient with preexisting autoimmune susceptibility.

## Case presentation

A 68-year-old Caucasian woman was referred to our endocrinology clinic in May 2022 following dual-energy X-ray absorptiometry demonstrating severe osteoporosis at multiple skeletal sites (Table 1). Her medical history was notable for Hashimoto thyroiditis, prior cholecystectomy in 2020, and no prior liver disease or alcohol use. No other autoimmune conditions were reported.

**Table 1:** DEXA scan results at baseline (March 2022) and after 2 years of denosumab therapy (June 2024)

Site	BMD 2022 (g/cm^2^)	*T*-score 2022	BMD 2024 (g/cm^2^)	T-score 2024
L1-L4	0.884	−2.5	0.932	−2.1
L1-L2	0.855	−2.6	0.892	−2.3
Left femoral neck	0.586	−3.3	0.637	−2.9
Right femoral neck	0.596	−3.2	0.615	−3.0
Left total hip	0.649	−2.9	0.694	−2.5
Right total hip	0.633	−3.0	0.670	−2.7
Left distal radius	0.225	−5.3	0.243	−4.9

BMD reported in g/cm^2^ (internationally accepted standard; no SI conversion applicable). *T*-scores below −2.5 are diagnostic of osteoporosis per World Health Organization criteria.

Abbreviations: BMD, bone mineral density; DEXA, dual-energy X-ray absorptiometry.

Baseline evaluation was unremarkable except for an elevated anti-thyroid peroxidase antibody at 41 IU/mL (reference <35 IU/mL). Baseline liver function tests were normal. Denosumab 60 mg subcutaneous injection was initiated in June 2022 every 6 months. Follow-up dual-energy X-ray absorptiometry in June 2024 demonstrated improvement across all sites (Table 1).

In April 2025, 3 years after denosumab initiation and following her seventh dose, the patient presented with jaundice, decreased appetite, fatigue, and dark urine. She was admitted from April 25 to May 2, 2025. Admission values included alanine aminotransferase (ALT) 2291 U/L (SI [International System of Units]: 38.3 µkat/L; reference <35 U/L [SI: <0.58 µkat/L]), aspartate aminotransferase (AST) 2101 U/L (SI: 35.1 µkat/L; reference <35 U/L [SI: <0.58 µkat/L]), alkaline phosphatase (ALP) 325 U/L (SI: 5.43 µkat/L; reference 44 to 147 U/L [SI: 0.73-2.45 µkat/L]), and total bilirubin 11.5 mg/dL (SI: 197 µmol/L; reference 0.2-1.2 mg/dL [SI: 3.4-20.5 µmol/L]). International Normalized Ratio was 1.3 (reference 0.8-1.2) and albumin 3.3 g/dL (SI: 33 g/L; reference 3.5-5.0 g/dL [SI: 35-50 g/L]).

## Diagnostic assessment

Comprehensive evaluation excluded alternative etiologies (Table 2). Abdominal magnetic resonance imaging and ultrasound showed no biliary obstruction or mass lesions. Viral hepatitis serologies—including hepatitis A, B, C, and E, Epstein–Barr virus, cytomegalovirus, and herpes simplex virus—were negative. Hemochromatosis, Wilson disease, and α-1 antitrypsin deficiency were excluded.

**Table 2:** Autoimmune hepatitis diagnostic workup (inpatient, April-May 2025)

Test	Result (conventional)	Result (SI)	Ref. range (conventional)	Ref. range (SI)
ANA	Positive	—	Negative	—
ASMA	39 Units (moderate to strong positive; LabCorp ELISA #006643)	—	Negative: 0-19 Units; weak positive: 20-30 Units; moderate to strong positive: >30 Units	—
AMA	Negative	—	Negative	—
Serum IgG	1552 mg/dL	SI: 15.52 g/L	700-1600 mg/dL	SI: 7.0-16.0 g/L
IL-2 receptor α chain (sCD25)	1932 U/mL	1932 U/mL	223-710 U/mL	223-710 U/mL
Hepatitis B surface antigen	Negative	—	Negative	—
Anti-HCV	Negative	—	Negative	—
CMV antibody	Negative	—	Negative	—
EBV antibody	Negative	—	Negative	—
HSV 1 and 2 antibody	Negative	—	Negative	—
Alpha-1 antitrypsin	169 mg/dL	SI: 1.69 g/L	101-187 mg/dL	SI: 1.01-1.87 g/L
Ceruloplasmin	Within normal limits	—	—	—
Liver biopsy	Chronic hepatitis suggestive of AIH; moderate interface hepatitis; lymphoplasmacytic portal infiltrate; many acidophil bodies; marked Kupffer cell hyperplasia; no advanced fibrosis	—	—	—
Peak ALT	2291 U/L	SI: 38.3 µkat/L	<35 U/L	SI: <0.58 µkat/L
Peak AST	2101 U/L	SI: 35.1 µkat/L	<35 U/L	SI: <0.58 µkat/L
Peak total bilirubin	25.2 mg/dL	SI: 431 µmol/L	0.2-1.2 mg/dL	SI: 3.4-20.5 µmol/L

Conversion factors: ALT/AST ×0.0167 (U/L → SI: µkat/L); bilirubin ×17.1 (mg/dL → SI: µmol/L); IgG and α-1 antitrypsin ×0.01 (mg/dL → SI: g/L). sCD25 reported in U/mL (LabCorp Interleukin-2 Soluble Receptor α ICMA, test #142455; reference 223-710 U/mL), directly comparable to the HLH-2004 diagnostic threshold of ≥2400 U/mL.

Abbreviations: AIH, autoimmune hepatitis; ANA, antinuclear antibody; ASMA, anti-smooth muscle antibody; AU/mL = arbitrary units per milliliter (ASMA reported in Units per LabCorp ELISA assay #006643); AMA, anti-mitochondrial antibody; ALT, alanine aminotransferase; AST, aspartate aminotransferase; CMV, cytomegalovirus; EBV, Epstein–Barr virus; HCV, hepatitis C virus; HSV, herpes simplex virus; IgG, immunoglobulin G; sCD25, soluble interleukin-2 receptor α chain (CD25).

Autoimmune evaluation revealed positive antinuclear antibodies and anti-smooth muscle antibodies of 39 Units (moderate to strong positive; reference: negative 0-19 Units, weak positive 20-30 Units, moderate to strong positive >30 Units; LabCorp ELISA, test #006643), and serum immunoglobulin G (IgG) of 1552 mg/dL (SI: 15.52 g/L; reference 700-1600 mg/dL [SI: 7.0-16.0 g/L]). Soluble interleukin-2 receptor α chain (sCD25) was 1932 U/mL (reference 223-710 U/mL; LabCorp ICMA, test #142455) and ferritin peaked at 6324 ng/mL (SI: 6324 µg/L; reference 11-307 ng/mL [SI: 11-307 µg/L]). Anti-mitochondrial antibody was negative.

Liver biopsy demonstrated moderate interface hepatitis, lymphoplasmacytic portal infiltrate, many acidophil bodies, and marked Kupffer cell hyperplasia with periodic acid-Schiff with diastase-positive staining—findings consistent with AIH. The pathologist noted the Kupffer cell hyperplasia raised the possibility of a hemophagocytic process such as hemophagocytic lymphohistiocytosis (HLH). Hematology and oncology consultation identified only 1 of 8 HLH-2004 criteria (hyperferritinemia: ferritin ≥500 ng/mL), well below the diagnostic threshold of 5, and HLH was excluded. The sCD25 value of 1932 U/mL (LabCorp ICMA, test #142455; reference 223-710 U/mL) did not meet the HLH-2004 diagnostic threshold of ≥2400 U/mL on the same assay platform and was therefore not counted as a met criterion.

The International AIH Scoring System yielded an aggregate score of 17, consistent with definite AIH. A Roussel Uclaf Causality Assessment Method (RUCAM) score of 7 suggested a probable drug association. However, RUCAM has recognized limitations in autoimmune-like liver injury, and the unusually long 3-year latency warrants cautious interpretation.

## Treatment

Prednisone was initiated during hospitalization, followed by azathioprine 100 mg daily and budesonide 9 mg daily (added July 2025, subsequently tapered). Denosumab was discontinued in June 2025. To prevent rebound bone loss, the patient was transitioned to intravenous zoledronic acid 5 mg annually in July 2025 [6].

## Outcome and follow-up

Serial liver function tests improved steadily (Table 3). By July 2025, ALT decreased to 43 U/L (SI: 0.72 µkat/L) and AST to 67 U/L (SI: 1.12 µkat/L). Full biochemical remission was achieved by January 2026: ALT 7 U/L (SI: 0.12 µkat/L), AST 18 U/L (SI: 0.30 µkat/L), ALP 61 U/L (SI: 1.02 µkat/L; reference 44-147 U/L [SI: 0.73-2.45 µkat/L]), and albumin 4.0 g/dL (SI: 40 g/L). The patient tolerated zoledronic acid without complication and continues azathioprine 100 mg daily. The requirement for long-term immunosuppression, rather than resolution with drug withdrawal alone, is itself more characteristic of idiopathic AIH than DI-ALH.

**Table 3:** Serial liver function test results during treatment

Date	ALT	AST	Alk Phos	Bilirubin	Albumin	INR	Ferritin
April 25, 2025 (admission)	2291 U/L (SI: 38.3 µkat/L)	2101 U/L (SI: 35.1 µkat/L)	325 U/L (SI: 5.43 µkat/L)	11.5 mg/dL (SI: 197 μmol/L)	—	1.3	—
May 7, 2025	1334 U/L (SI: 22.3 µkat/L)	615 U/L (SI: 10.3 µkat/L)	186 U/L (SI: 3.11 µkat/L)	25.2 mg/dL (SI: 431 μmol/L)	3.3 g/dL (SI: 33 g/L)	—	6,324 ng/mL (SI: 6,324 µg/L)
May 9, 2025	767 U/L (SI: 12.8 µkat/L)	351 U/L (SI: 5.86 µkat/L)	195 U/L (SI: 3.26 µkat/L)	23.4 mg/dL (SI: 400 μmol/L)	3.4 g/dL (SI: 34 g/L)	—	—
July 2025	43 U/L (SI: 0.72 µkat/L)	67 U/L (SI: 1.12 µkat/L)	135 U/L (SI: 2.26 µkat/L)	0.7 mg/dL (SI: 12.0 μmol/L)	3.9 g/dL (SI: 39 g/L)	1.0	—
August 2025	15 U/L (SI: 0.25 µkat/L)	25 U/L (SI: 0.42 µkat/L)	67 U/L (SI: 1.12 µkat/L)	—	3.7 g/dL (SI: 37 g/L)	—	—
October 2025	11 U/L (SI: 0.18 µkat/L)	25 U/L (SI: 0.42 µkat/L)	56 U/L (SI: 0.94 µkat/L)	—	4.3 g/dL (SI: 43 g/L)	—	—
January 2026	7 U/L (SI: 0.12 µkat/L)	18 U/L (SI: 0.30 µkat/L)	61 U/L (SI: 1.02 µkat/L)	—	4.0 g/dL (SI: 40 g/L)	—	—

Values shown as conventional (SI: value). Conversion factors: ALT, AST, Alk Phos ×0.0167 (U/L → SI: µkat/L); bilirubin ×17.1 (mg/dL → SI: µmol/L); albumin ×10 (g/dL → SI: g/L); ferritin: ng/mL = SI: µg/L (numerically identical). Normal ranges: ALT <35 U/L (SI: <0.58 µkat/L); AST <35 U/L (SI: <0.58 µkat/L); Alk Phos 44 -147 U/L (SI: 0.73 -2.45 µkat/L); bilirubin 0.2 -1.2 mg/dL (SI: 3.4 -20.5 µmol/L); albumin 3.5 -5.0 g/dL (SI: 35 -50 g/L); INR 0.8 -1.2; ferritin (female) 11 -307 ng/mL (SI: 11 -307 µg/L).

Abbreviations: ALT, alanine aminotransferase; AST, aspartate aminotransferase; Alk Phos, alkaline phosphatase; INR, International Normalized Ratio.

## Discussion

This case presents a diagnostic challenge: severe AIH arising after 3 years of denosumab therapy in a patient with preexisting Hashimoto thyroiditis. Two nonmutually exclusive diagnoses must be weighed: (1) denosumab-associated DI-ALH and (2) de novo idiopathic AIH in a patient with underlying autoimmune susceptibility. The distinction carries important prognostic implications—DI-ALH typically resolves with drug withdrawal and a short corticosteroid course, whereas idiopathic AIH requires lifelong immunosuppression. The need for ongoing immunosuppressive therapy is more consistent with idiopathic autoimmune hepatitis, although prolonged treatment may also be appropriate in selected patients with severe drug-induced autoimmune-like hepatitis.

The biopsy findings also merit discussion. Classic AIH features, such as moderate interface hepatitis and lymphoplasmacytic infiltrate, were present alongside marked Kupffer cell hyperplasia with periodic acid-Schiff with diastase–positive staining, prompting pathologist concern for a hemophagocytic process. Hemophagocytic lymphohistiocytosis is a hyperinflammatory syndrome requiring ≥5 of 8 HLH-2004 criteria; our patient met only 1 confirmed criterion (hyperferritinemia: ferritin 6324 ng/mL [SI: 6324 µg/L]). The sCD25 of 1932 U/mL (LabCorp ICMA, test #142455) was elevated above the laboratory reference range of 223 to 710 U/mL but did not meet the HLH-2004 diagnostic threshold of ≥2400 U/mL on the same assay platform. The absence of cytopenias, splenomegaly, hypertriglyceridemia, or confirmed hemophagocytosis on biopsy, and the robust response to corticosteroids without HLH-directed therapy support AIH as the primary diagnosis.

The case for a drug-associated mechanism rests on a RUCAM score of 7, the absence of prior liver disease despite decades of autoimmune predisposition, and biological plausibility through RANKL's immunomodulatory role on Kupffer cells and lymphocyte subsets [7, 8]. At least 3 cases of denosumab-associated hepatic injury have been published, including submassive necrosis [9] and 2 cases of AIH, both in patients with concurrent autoimmune conditions [4, 5, 9]. However, a 3-year latency is substantially longer than the latency reported in most published cases of drug-induced autoimmune-like hepatitis [10]. A possible drug-associated mechanism would involve prolonged RANKL blockade progressively disrupting hepatic immune tolerance—through effects on Kupffer cells and regulatory T cells—until a threshold is crossed and overt liver injury develops. However, no hepatotoxicity signal was reported in the 10-year FREEDOM Extension trial despite long-term follow-up [11]. Furthermore, a large-scale disproportionality analysis of the FDA Adverse Event Reporting System database identifying 50 drugs with strong DI-ALH signals, including nitrofurantoin, minocycline, and several biologics, did not identify denosumab as a drug significantly associated with DI-ALH risk [12].

The presence of Hashimoto thyroiditis increases the pretest probability of idiopathic AIH. Hashimoto thyroiditis is the most common concurrent autoimmune disease in type 1 AIH, present in 10% to 23% of patients; in patients aged ≥60 years, concurrent autoimmune thyroid disease is present in up to 42%—compared with 13% in younger adults [3]. This patient's clinical profile is entirely consistent with idiopathic AIH emerging in a genetically susceptible individual, with denosumab as an incidental exposure or possible environmental trigger.

Regardless of mechanism, this case illustrates that significant hepatic injury, whether drug-associated or idiopathic, can occur in patients on denosumab. Clinicians should not dismiss transaminase elevation in this setting. The International AIH Score of 17 (Table 4) and the HLH-2004 criteria evaluation (Table 5) provided structured diagnostic frameworks independent of the etiologic question. Following denosumab discontinuation, prompt bisphosphonate transition is essential to prevent rebound bone loss [6]. Because rechallenge with denosumab was neither clinically appropriate nor ethically justified, definitive attribution of causality is not possible.

**Table 4:** International Autoimmune Hepatitis Score (Alvarez et al)

Parameter (patient value)	Criterion satisfied	Points
Female sex	Yes	+2
ALP:AST ratio (325 ÷ 2101 = 0.155; <1.5)	Yes	+2
Serum IgG (1552 mg/dL; within normal range)	No	0
ANA/SMA (ASMA 39 Units [moderate to strong positive >30 Units]; LabCorp ELISA, test #006643; ANA positive by immunofluorescence; no IIF titer available)	Yes	+2
Illicit drug use history	Yes	+1
Average alcohol intake daily	Yes	+2
Histological findings: moderate interface hepatitis; lymphoplasmacytic infiltrate	Yes	+3
Other autoimmune disease (Hashimoto thyroiditis)	Yes	+2
AMA positivity	No	0
Hepatitis viral markers (all negative)	Yes	+3
**Aggregate score**		**17**
**Interpretation**	**Definite AIH (>15)**	

Definite AIH: >15; Probable AIH: 10-15. ASMA measured by LabCorp ELISA (test #006643, Actin [Smooth Muscle] Antibody); result reported in Units (reference range: negative 0-19; weak positive 20-30; moderate to strong positive >30). Patient result of 39 Units falls in the moderate to strong positive category. Because this assay uses ELISA rather than indirect immunofluorescence (IIF), a formal titer cannot be derived. For ELISA-based ASMA assays, a clearly positive result above the assay's reference range is considered diagnostically meaningful per the revised International AIH Scoring System; the autoantibody criterion is therefore scored at +2. ANA was positive by immunofluorescence; formal dilutional titer not available. If ANA titer ≥1:80 is confirmed, aggregate score increases to 18. If the autoantibody criterion is conservatively scored as 0, the aggregate score is 15, consistent with Probable AIH (10-15), which remains clinically significant and does not alter the conclusions of this case.

Abbreviations: AIH, autoimmune hepatitis; ALP, alkaline phosphatase; AMA, anti-mitochondrial antibody; ANA, antinuclear antibody; ASMA, anti-smooth muscle antibody; AST, aspartate aminotransferase; AU/mL, arbitrary units per milliliter.

**Table 5:** HLH-2004 diagnostic criteria applied to this patient

HLH-2004 criterion	Diagnostic threshold	Patient value/finding	Criterion satisfied
Fever (≥38.5 °C)	—	Not documented	No
Splenomegaly	—	Not documented on imaging	No
Cytopenias (≥2 cell lines)	Hgb <9 g/dL (SI: <90 g/L); Plt <100 × 10^9^/L; ANC <1.0 × 10^9^/L	Plt 153 × 10^9^/L; no cytopenias	No
Hypertriglyceridemia (≥265 mg/dL [SI: ≥2.99 mmol/L]) and/or hypofibrinogenemia (≤150 mg/dL [SI: ≤1.5 g/L])	—	Not met per hematology/oncology	No
Hemophagocytosis on biopsy	—	Not confirmed; Kupffer cell hyperplasia noted	No
Low/absent NK cell activity	—	Not met per hematology/oncology	No
Ferritin ≥500 ng/mL (SI: ≥500 µg/L)	Ferritin ≥500 ng/mL	6324 ng/mL (SI: 6324 µg/L)	Yes
Elevated sCD25 ≥ 2400 U/mL	sCD25 ≥ 2400 U/mL	1932 U/mL (above reference range of 223-710 U/mL; does not meet HLH-2004 threshold of ≥2400 U/mL on same assay platform)	Not confirmed
**TOTAL CRITERIA MET**		**1 confirmed of 8**	**HLH excluded (≥5 required)**

Diagnosis requires ≥5 of 8 criteria. Patient met 1 confirmed criterion of 8; HLH excluded following hematology and oncology consultation. sCD25 measured by LabCorp Interleukin-2 Soluble Receptor α ICMA (test #142455). Result of 1932 U/mL is above the assay reference range (223-710 U/mL) but does not meet the HLH-2004 diagnostic threshold of ≥2400 U/mL on the same assay platform; it was therefore not counted as a met criterion. Ferritin ng/mL = SI: µg/L (numerically identical).

Abbreviations: ANC, absolute neutrophil count; HLH, hemophagocytic lymphohistiocytosis; Hgb, hemoglobin; NK = natural killer; Plt, platelets; sCD25, soluble interleukin-2 receptor α chain (CD25).

## Learning points

- Autoimmune hepatitis should be considered in patients receiving denosumab who develop jaundice or marked transaminase elevation, although distinguishing DI-ALH from de novo idiopathic AIH may be challenging.
- A prolonged latency of 3 years after denosumab initiation is atypical for drug-induced autoimmune-like hepatitis and should prompt consideration of underlying idiopathic AIH, particularly in patients with preexisting autoimmune disease.
- The revised International Autoimmune Hepatitis Scoring System can support diagnosis, while structured application of the HLH-2004 criteria helps exclude HLH in patients with hyperferritinemia and atypical biopsy findings.
- Following denosumab discontinuation, prompt transition to bisphosphonate therapy is essential to prevent rebound bone loss while immunosuppressive therapy is initiated for autoimmune hepatitis

## Contributors

J.M. conceptualized the manuscript, performed the literature review, and drafted and submitted the manuscript. M.A. was involved in the clinical diagnosis and management of the patient, contributed to data interpretation, and critically revised the manuscript. A.M. contributed to the clinical management of the patient and provided critical revision of the manuscript for important intellectual content. All authors gave final approval of the version to be published and agree to be accountable for all aspects of the work.

## Data Availability

Original data generated and analyzed during this study are included in this published article.

## Abbreviations

AIH: autoimmune hepatitis
ALP: alkaline phosphatase
ALT: aminotransferase
AMA: anti-mitochondrial antibody
ANA: aminotransferase
ANC: absolute neutrophil count
ASMA: anti-smooth muscle antibody
AST: aminotransferase
BMD: bone mineral density
CMV: cytomegalovirus
DEXA: dual-energy X-ray absorptiometry
DI: distinguishing
DI-ALH: drug-induced autoimmune-like hepatitis
EBV: Epstein–Barr virus
HLH: hemophagocytic lymphohistiocytosis
HSV: herpes simplex virus
IIF: indirect immunofluorescence
INR: International Normalized Ratio
NK: natural killer
RANKL: receptor activator of nuclear factor-κB ligand
RUCAM: Roussel Uclaf Causality Assessment Method
sCD25: soluble interleukin-2 receptor α chain
